# Impact of an Adenosine A_2A_ Receptor Agonist and Antagonist on Binding of the Dopamine D_2_ Receptor Ligand [^11^C]raclopride in the Rodent Striatum

**DOI:** 10.1021/acs.molpharmaceut.2c00450

**Published:** 2022-07-18

**Authors:** Kavya Prasad, Erik F. J. de Vries, Jürgen W. A. Sijbesma, Lara Garcia-Varela, Daniel A. Vazquez-Matias, Rodrigo Moraga-Amaro, Antoon T. M. Willemsen, Rudi A. J. O. Dierckx, Aren van Waarde

**Affiliations:** Department of Nuclear Medicine and Molecular Imaging, University Medical Center Groningen, University of Groningen, Hanzeplein 1, P.O. Box 30001, 9713 GZ Groningen, The Netherlands

**Keywords:** A_2A_ receptor, D_2_ receptor, animal studies, kinetic modeling, positron emission tomography

## Abstract

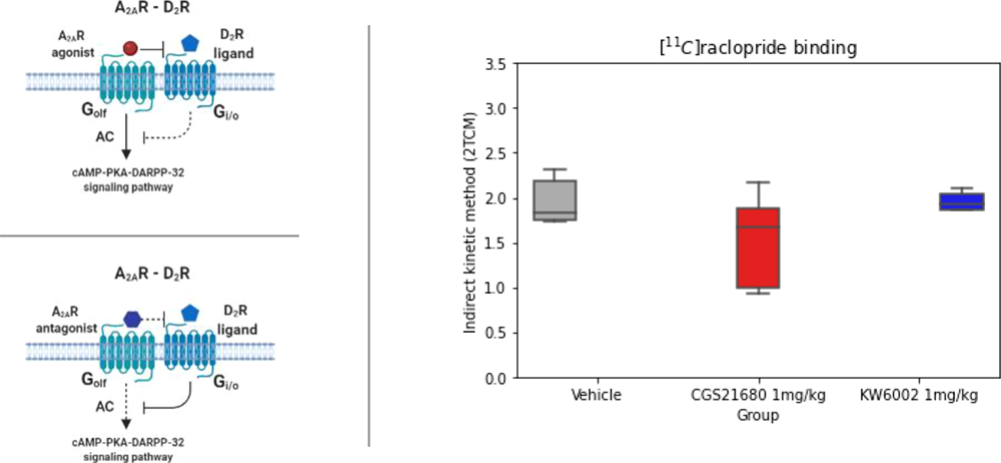

Adenosine A_2A_ and dopamine D_2_ receptors in the basal ganglia form heterotetrameric structures that are involved in the regulation of motor activity and neuropsychiatric functions. The present study examines the A_2A_ receptor-mediated modulation of D_2_ receptor binding in vivo using positron emission tomography (PET) with the D_2_ antagonist tracer [^11^C]raclopride. Healthy male Wistar rats (*n* = 8) were scanned (60 min dynamic scan) with [^11^C]raclopride at baseline and 7 days later following an acute administration of the A_2A_ agonist CGS21680 (1 mg/kg), using a MicroPET Focus-220 camera. Nondisplaceable binding potential (BP_ND_) values were calculated using a simplified reference tissue model (SRTM), with cerebellum as the reference tissue. SRTM analysis did not show any significant changes in [^11^C]raclopride BP_ND_ (*p* = 0.102) in striatum after CGS21680 administration compared to the baseline. As CGS21680 strongly affects hemodynamics, we also used arterial blood sampling and a metabolite-corrected plasma input function for compartment modeling using the reversible two-tissue compartment model (2TCM) to obtain the BP_ND_ from the *k*_3_/*k*_4_ ratio and from the striatum/cerebellum volume of distribution ratio (DVR) in a second group of animals. These rats underwent dynamic [^11^C]raclopride scans after pretreatment with a vehicle (*n* = 5), a single dose of CGS21680 (1 mg/kg, *n* = 5), or a single dose of the A_2A_ antagonist KW6002 (1 mg/kg, *n* = 5). The parent fraction in plasma was significantly higher in the CGS21680-treated group (*p* = 0.0001) compared to the vehicle-treated group. GCS21680 administration significantly reduced the striatal *k*_3_/*k*_4_ ratio (*p* < 0.01), but *k*_3_ and *k*_4_ estimates may be less reliable. The BP_ND_ (DVR-1) decreased from 1.963 ± 0.27 in the vehicle-treated group to 1.53 ± 0.55 (*p* = 0.080) or 1.961 ± 0.11 (*p* = 0.993) after the administration of CGS21680 or KW6002, respectively. Our study suggests that the A_2A_ agonist CGS21680, but not the antagonist KW6002, may reduce the D_2_ receptor availability in the striatum.

## 1 Introduction

**Figure 1:**
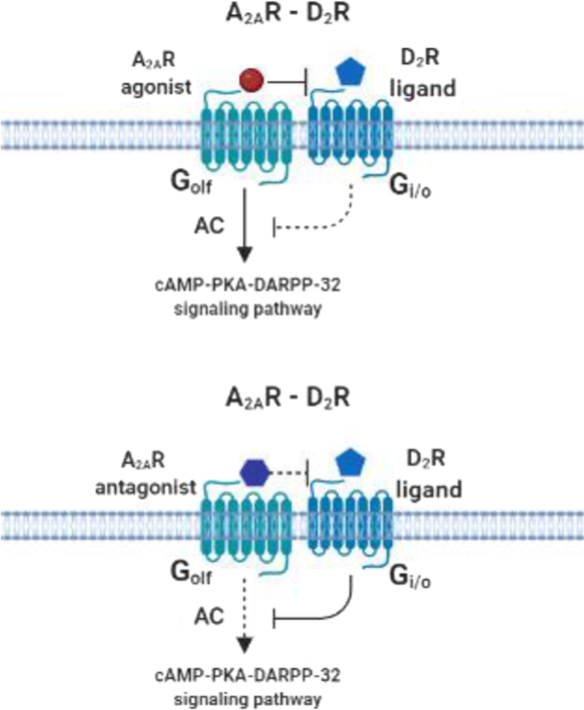
Schematic representation of A_2A_–D_2_ heteromers. At the intramembrane level, antagonistic interactions take place, and the two receptors cause opposite effects on the signal cascade mediated by adenylyl cyclase (AC). Created with BioRender.com.

Adenosine is a neuromodulator and a metabolite of adenosine triphosphate (ATP) that plays several behavioral and physiological roles throughout the central nervous system via interaction with multiple receptors. Adenosine receptors (AR) are G-protein-coupled proteins with four known subtypes called A_1_R, A_2A_R, A_2B_R, and A_3_R which are widely distributed in several regions of the brain.^1^ These GPCRs form homodimers and heteromers, which are involved in cell signaling. The A_2A_R–D_2_R heteromer plays an important role in the modulation of GABAergic striatopallidal neuronal functions. Administration of agonists and antagonists of A_2A_R can result in the conformational changes of the heteromer complex. These changes cause a modification in the affinity of D_2_R toward its own ligands^2^ (Figure 1). This receptor interaction results in the modulation of neuronal excitability and neurotransmitter release. The most notable regulatory functions of the A_2A_R–D_2_R heterotetrameric complex in the mammalian brain include control of locomotion, anxiety, cognition, and memory.^3^ Shifts of the homomer/heteromer equilibrium, altered expression, or altered function of the receptors in the heteromer have been associated with motor and cognitive disturbances in neurological disorders.

In rodent studies, A_2A_R agonists, such as CGS21680, have been shown to play a neuroleptic role when administered systemically at low doses. A_2A_R agonists are associated with sedation and/or drowsiness, and their actions are similar to those of the antagonists of D_2_R.^4^ On the other hand, A_2A_R antagonists facilitate the motor-activating effects of dopamine agonists.^5^ A_2A_R–D_2_R heteromers are considered as the targets for drug treatment, as they are involved in the modulation of dopaminergic, glutamatergic, and GABAergic neurotransmission. Autoradiography experiments have provided support for the antagonistic and allosteric interactions between A_2A_R and D_2_R within A_2A_R–D_2_R heteromers in the striatal sections of both rat and human brain. In such sections, CGS21680 decreased the ability of dopamine to displace the bound D_2_/D_3_ antagonist [^125^I]iodosulpiride.^6^

In vivo receptor-binding studies that indicate altered D_2_R functions upon administration with A_2A_ agonists have not been reported, although such functional changes could be demonstrated using PET imaging. The present study aims to determine the effects of a specific A_2A_R agonist and an A_2A_R antagonist on the regional availability of D_2_R’s using PET with the dopamine D_2_ receptor ligand [^11^C]raclopride. [^11^C]raclopride is a validated tracer for dopamine D_2_/D_3_ receptors in the striatum.^7,8^ The equilibrium dissociation constant *K*_d_*V*_R_ value of [^11^C]raclopride obtained by compartmental modeling was 6.2 nmol/L, and the affinity (*K*_d_) by equilibrium analysis was 10 nM.^8^ CGS21680 is considered as a potent A_2A_R agonist that is 100 times selective for A_2A_ over A_1_ receptors and is capable of crossing the blood–brain barrier. It binds to the A_2A_ receptor with high affinity (*K*_d_ = 15.5 nM).^9^ KW6002 is considered as a selective A_2A_R antagonist whose affinity for the A_2A_ receptor is 9.12 nM.^10^ As adenosine agonists are vasodilator drugs that are hypotensive, we verified the doses administered. A very high dose of CGS21680 (10 mg/kg) induces pronounced peripheral side effects such as tachycardia and diarrhea. Thus, we used a 10-fold lower dose that does not cause such peripheral effects.^11^ The preferred dose was still high enough to saturate receptors, a method that remains as a gold standard for occupancy in PET imaging. On the other hand, KW6002 does not have potent peripheral effects. The strength of adenosine–dopamine interactions in the living brain could be determined by measuring the changes of the binding of [^11^C]raclopride to D_2_R after the stimulation or blockade of A_2A_R’s using an A_2A_R agonist or antagonist.^12^

## 2 Methods

### 2.1 Experimental Animals

**Figure 2:**
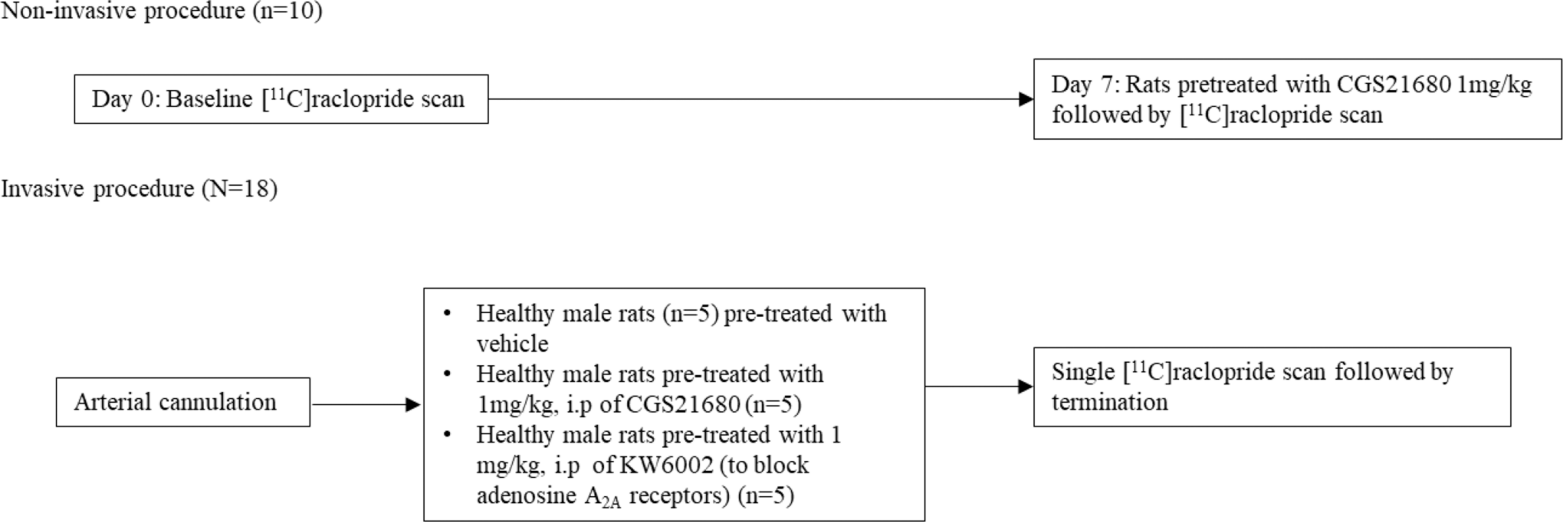
Scheme showing the study design: noninvasive and invasive experimental procedure.

Male outbred Wistar rats (*n* = 28 Hsd/Cpb:WU, 10–12 weeks old, 300–400 g) were purchased from Envigo (the Netherlands). The experiments were approved by the Dutch National Committee on Animal Experiments (CCD: AVD1050020198648) and the Institutional Animal Care and Use Committee of the University of Groningen (IvD 15166-01-004 and IvD: 198648–01-002). The rats were housed in groups in humidity- and temperature-controlled rooms (21 ± 2 °C) with a 12 h light–dark cycle. Animals were fed with standard laboratory chow and water ad libitum. They were allowed to acclimatize for at least 7 days after their arrival from the supplier. Animals were divided in two cohorts according to the applied scanning protocol: noninvasive and invasive (without or with arterial blood sampling). Noninvasive D_2_R PET imaging was performed in 10 animals, using a reference tissue model for the quantification of tracer binding. Rats were scanned at baseline (without pretreatment) and were scanned again within 7 days, after a drug challenge (pretreatment with CGS21680 (1 mg/kg)). Invasive D_2_R imaging with arterial blood sampling was done once in animals randomly divided into three groups (*n* = 5) that were treated with, respectively, a vehicle (31% polyethylene glycol (PEG 400) and 0.5% dimethyl sulfoxide (DMSO) in saline), an A_2A_ agonist CGS21680 (1 mg/kg in a solution of 31% PEG 400 and 0.5% DMSO in saline), or an A_2A_ antagonist KW6002 (1 mg/kg in a solution of 31% PEG 400 and 0.5% DMSO in saline) (Figure 2). The vehicle and A_2A_ ligands were administered 10 min before the injection of the radiotracer for PET imaging, so that the ligands could reach the A_2A_R/D_2_R heteromers earlier than the radiotracer. Heart rate and blood oxygenation of the animals were monitored throughout the experiment, using pulse oximeters. We were forced to exclude data of two rats from the noninvasive study and three rats from the invasive study (one vehicle-treated and two KW6002-treated) due to improper execution of the study protocol (*n* = 2) or untimely death of the animals (*n* = 3). We compensated for the loss of rats from the invasive study by ordering extra animals and performing additional scans.

### 2.2 PET Imaging

Prior to PET imaging, animals were anesthetized with isoflurane in oxygen (5% isoflurane for induction and 1.5–2.5% isoflurane for maintenance). Eye salve was applied to prevent dehydration of the cornea. The animals included in the noninvasive studies (*n* = 10) were cannulated in a tail vein for the injection of [^11^C]raclopride before the baseline and follow-up scans. Rats were injected intraperitoneally with CGS21680 (1 mg/kg) 10 min before the injection of the PET tracer and start of the follow-up scan.

The animals included in the invasive studies (*n* = 18) were cannulated in a tail vein (for the injection of [^11^C]raclopride), followed by insertion of a second cannula in a femoral artery for arterial blood sampling. They were injected intraperitoneally with the vehicle, CGS21680 (1 mg/kg) or KW6002 (1 mg/kg) in a total volume of 1 mL, 10 min before the injection of [^11^C]raclopride. Pretreatment of animals with a high dose of a specific (nonradioactive) receptor ligand is the standard method used in PET imaging to assess whether a PET tracer binds to its intended target. The specific agonist and antagonist used in this protocol (CGS21680 and KW6002) have been shown to be well tolerated by rats after intraperitoneal administration (in doses up to 3 mg/kg), to enter the brain and to exert central effects. Only one PET scan was made in animals subjected to arterial blood sampling. Body weight (328 ± 11 g) was determined before the start of the scan.

PET images were acquired using a Focus 220 MicroPET camera (Preclinical Solutions, Siemens Healthcare Molecular Imaging, Knoxville, Tennessee, USA Inc.). Two rats were scanned simultaneously, with their heads positioned in the field of view. A transmission scan with a ^57^Co point source was acquired for attenuation correction. The rats were intravenously injected with 25.0 ± 3.2 MBq [^11^C]raclopride (injected mass 0.81 ± 0.29 nmol; molar activity: 32.9 ± 7.7 MBq/nmol) for noninvasive studies and with 32.2 ± 4.8 MBq [^11^C]raclopride (injected mass: 0.83 ± 0.25 nmol; molar activity: 41.3 ± 10.0 MBq/nmol) for invasive studies. The tracer was injected with an infusion pump at a speed of 1 mL/min for the first minute of the 60 min duration of dynamic acquisition. Heart rate and oxygen saturation were monitored at regular (10 min) intervals. Body temperature was maintained between 35 and 37 °C throughout the scan by the use of heating pads.

PET data were corrected for decay and attenuation. A 2D OSEM (ordered subset maximization algorithm) reconstruction, followed by Fourier rebinning, was used for iterative reconstructions. The emission sinograms were used for iterative reconstructions involving 4 iterations and 16 subsets. This was followed by a list-mode data binning into 21 frames (6 × 10, 4 × 30, 2 × 60, 1 × 120, 1 × 180, 4 × 300, 3 × 600 s) and an image matrix of 256 × 256 × 95 pixels with a slice thickness of 0.796 mm and a pixel width of 0.633 mm.

### 2.3 Arterial Sampling and Metabolite Analysis

Blood samples with a volume of 0.10–0.13 mL were drawn from the femoral artery at 10, 20, 30, 40, 50, 60, and 90 s and 2, 3, 5, 7.5, 10, 15, 30, and 60 min after tracer administration. After the collection of each sample, an equal volume of saline with 1% heparin was infused into the artery to compensate for the blood volume loss. Larger blood samples (0.8–1 mL) were drawn at 5, 10, 30, and 60 min for [^11^C]raclopride metabolite analysis. From each blood sample, a plasma sample was obtained by the centrifugation of whole blood for 5 min at 3000 g. The radioactivity in 25 μL whole blood and in 25 μL plasma was measured with an automated well counter (Wizard 2480, Perkin Elmer, USA) and was corrected for decay.

In the plasma samples obtained at 5, 10, 30, and 60 min after tracer injection, the fraction of radioactivity representing unchanged [^11^C]raclopride was determined by high-performance liquid chromatography (HPLC; Platinum C18 5 μ column (250x10mm), isocratic elution system, mobile phase: 25% ACN/75% water, pH = 2, acidified with HClO_4_, flow rate: 0.6 mL/min). Plasma (0.4 mL) was diluted with 0.4 mL of acetonitrile, vortexed, and centrifuged at 3000 g for 3 min. The resulting supernatant was passed through a Millipore HV filter. The sample was diluted with 0.5 mL of 0.1 M ammonium formate solution, after which the resulting mixture was analyzed by HPLC. Fractions of 30 s were collected and measured in the automated well counter. The percentage of unchanged [^11^C]raclopride in plasma was calculated by dividing radioactivity in the fractions corresponding to unchanged [^11^C]raclopride by the total radioactivity in the eluate and multiplying by 100%.

### 2.4 PET Data Analysis

The researchers were not blinded during the experiments, but data analysis was done using automated procedures and was thus operator-independent. Data analysis was performed using PMOD 4.0 software (PMOD Technologies, Zürich, Switzerland). The averaged PET images acquired between 40 and 60 min after tracer injection were aligned to a tracer-specific reference template for [^11^C]raclopride. The same transformation matrix was subsequently applied to dynamic PET frames in order to automatically co-register them to the reference template. A volume of interest (VOI) atlas containing the striatum and cerebellum was then placed on each co-registered PET image. Individual time–activity curves (TACs, in kBq/mL) were generated for each VOI from the dynamic data.

### 2.5 Pharmacokinetic Modeling

(1) For the noninvasive procedure, nondisplaceable binding potential (BP_ND_) values were calculated using the simplified reference tissue model (SRTM). The SRTM is a reference tissue model that characterizes the kinetic behavior of the radioligand in the target and reference regions by assuming that the influx-to-efflux ratio (*K*_1_/*k*_2_) is similar in all brain regions. The BP_ND_ at the target region is obtained using the following solution to the system.^14^

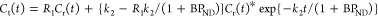
where *C*_t_(*t*) is the target tissue time activity curve, *C*_r_(*t*) is the reference tissue time activity curve, *R*_1_ is the relative rate of delivery of the tracer given by the target-to-reference *K*_1_ ratio (*K*_1_/*K*_1_′), and *k*_2_ is the efflux rate constant.

(2) For invasive procedures, the BP_ND_ was estimated using the two-tissue compartment model (2TCM). The 2TCM uses two distinct tissue compartments to calculate the kinetics of the radioligand. The differential equations that define the two compartments are
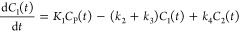

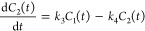
where *K*_1_ and *k*_2_ are the efflux and influx rate constants for the transfer of [^11^C]raclopride between plasma and brain, whereas *k*_3_ and *k*_4_ are the rate constants that define the exchange between the free and specifically bound radioligand pools in the tissue. *C*_P_(*t*) is the tracer concentration in plasma at time *t*. Metabolite-corrected arterial plasma and arterial whole-blood curves were used as inputs to fit 2TCM across regions. The BP_ND_ was estimated either by calculating the ratio of the rate constants *k*_3_ and *k*_4_ in the striatum (*k*_3_/*k*_4_) or by determining the volumes of distribution of [^11^C]raclopride (*V*_T_) in the striatum and cerebellum. The BP_ND_ can then be calculated from the *V*_T_ ratio (DVR) according to the formula^15^
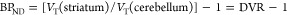


The standard error of the estimated microparameters (*K*_1_, *k*_2_, *k*_3_, and *k*_4_) was always less than 25%. A smaller percentage indicates better identifiability. Pearson *r* was used to assess correlations between the BP_ND_ values estimated from the 2TCM and SRTM. The cerebral blood volume component was fixed to 0.05 mL/cm^3^ for all models and brain regions.^13^

### 2.6 Statistical Analysis

Statistical analysis was performed using SPSS 23 and Python 3.8 software. The TACs were expressed as SUV. Differences between the area under the curve (AUC) for the parent fraction, whole blood, and plasma (with or without correction for metabolites) were examined using one-way analysis of variance (ANOVA). The BP_ND_ estimated from *k*_3_/*k*_4_ and DVR-1 and *V*_T_ and individual *K*_1_, *k*_2,_*k*_3_, and *k*_4_ values obtained from the 2TCM were analyzed using one-way ANOVA, with treatment as a between-group factor. Post hoc analysis for comparison between vehicle (control) and treatments was performed using the least significant difference (LSD) test. Differences were considered significant when the *p* value was <0.05. A paired *t* test was used to determine the differences between output parameters for animals belonging to the noninvasive group. The effect size, given by Cohen’s *d*, was estimated using G*Power software (Universities of Kiel, Düsseldorf, and Mannheim, Germany). For power calculations, the option “difference between two independent means, matched pairs” in the program was chosen. The required sample size for each group was calculated by comparing the means of control and treatment animals using *t* tests, alpha = 0.05, and power = 0.90. The effect size is defined as the mean difference between the vehicle group and each of the treated groups, divided by the pooled standard deviation.^16^ Values from Cohen’s *d* corresponding to 0.5, 0.8, 1.2, and 2 were considered to reflect medium, large, very large, and huge effects, respectively.^17^

## 3 Results

### 3.1 Brain Kinetics of [^11^C]raclopride from Rats Scanned at Baseline and at Follow-Up

**Figure 3:**
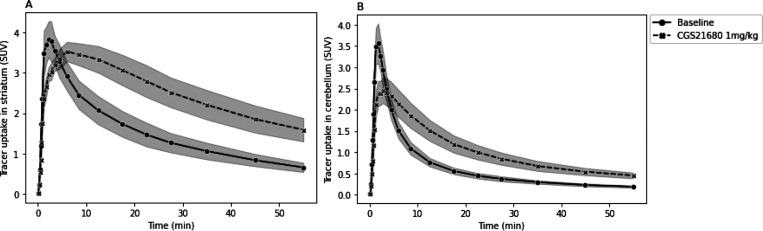
Time–activity curves of [^11^C]raclopride in the striatum (A) and cerebellum (B) of rats at baseline and after pretreatment with 1 mg/kg CGS21680 (data are expressed as mean ± SD).

In animals that underwent a baseline scan, followed by a post-dose scan after 7 days, the baseline striatal TACs showed a high initial peak uptake between 1 and 2 min after tracer injection. The TACs of follow-up after pretreatment with CGS21680 showed a delayed peak (obtained at 6 min) and higher values at later time points (Figure 3A).

### BP_ND_ from SRTM in Rats Scanned at Baseline and at Follow-Up

**Figure 4:**
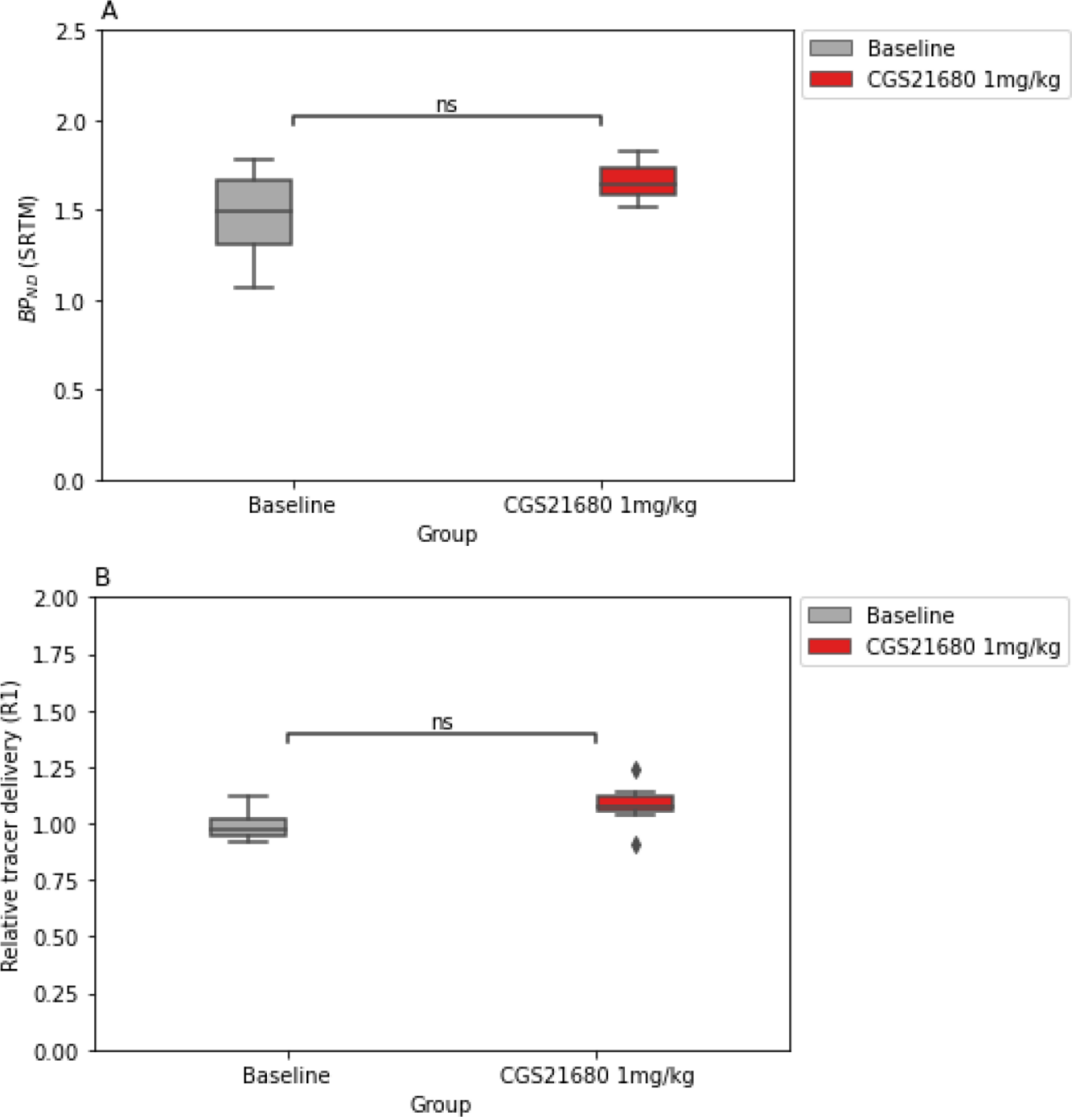
Binding potential and relative tracer delivery derived from the baseline and follow-up scans. (A) Nondisplaceable binding potential (BP_ND_) of the striatum (*p* = 0.102, Cohen’s *d* = 0.63). (B) Relative delivery ratio [*R*_1_ = *K*_1_/*K*_1_′] between the striatum and cerebellum (*p* = 0.054, Cohen’s *d* = 0.84).

Striatal BP_ND_ values obtained from SRTM revealed no significant differences between the PET scans that were acquired at baseline and the scans performed after pretreatment with CGS21680 (*n* = 8; *t* = 1.769, df = 7; *p* = 0.120, Cohen’s *d* = 0.63; Figure 4A). The relative tracer delivery *R*_1_, which is the ratio of *K*_1_ in the target tissue to *K*_1_′ in the reference tissue, was not significantly different between the baseline and follow-up scans (*t* = 2.31, df = 7; *p* = 0.054, Cohen’s *d* = 0.82; Figure 4B and Table S2).

### 3.2 Brain Kinetics of [^11^C]raclopride in PET Scans with Arterial Blood Sampling

**Figure 5:**
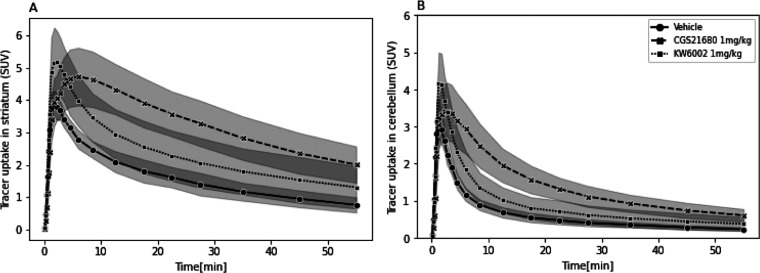
Time–activity curves of [^11^C]raclopride in (A) striatum and (B) cerebellum of vehicle-, CGS21680-, and KW6002-treated rats (data are expressed as mean ± SD).

In animals that underwent arterial blood sampling, the striatal TACs from rats that received vehicle or KW6002 treatment showed a high initial peak uptake between 1 and 2 min after tracer injection. The TACs of [^11^C]raclopride in the vehicle group had a lower initial peak than in the CGS21680- and KW6002-treated rats. The striatal TAC of rats that obtained CGS21680 treatment showed a delayed peak (obtained at 4.5 min) and higher values at later time points compared to the vehicle- and KW6002-treated groups (Figure 5A).

In the cerebellum, the highest uptake was also found between 1 and 2 min in the vehicle- and KW6002-treated rats. Peak values in these groups were reached earlier than in CGS21680-treated rats (peak at 3 min), as depicted in Figure 5B.

### BP_ND_ Derived from the SRTM in Rats Receiving PET Scans with Arterial Blood Sampling

**Figure 6:**
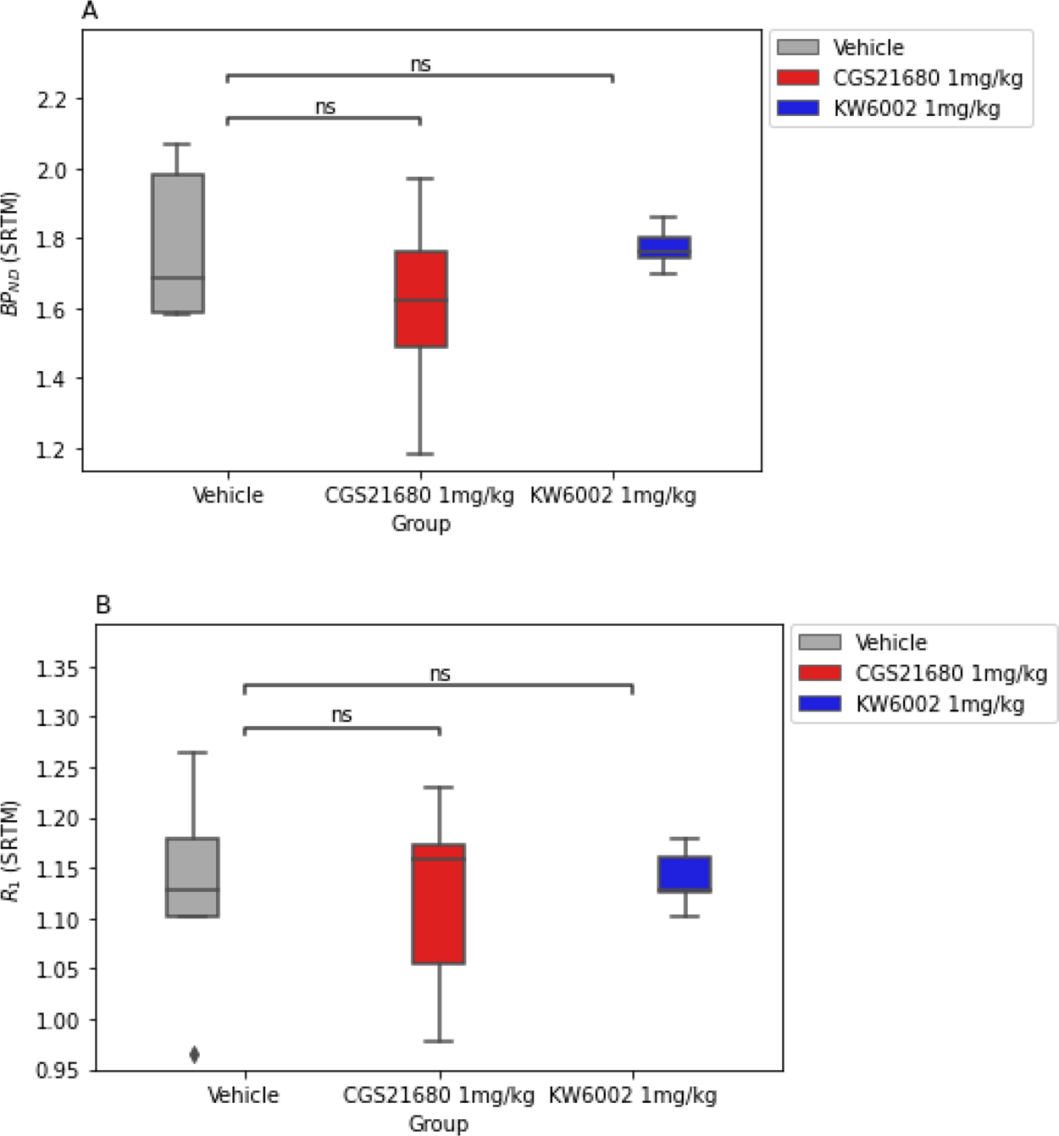
(A) Binding potential (BP_ND_) of [^11^C]raclopride in the striatum for vehicle-, CGS21680-, and KW6002-treated rats. (B) Relative delivery ratios [*R*_1_ = *K*_1_/*K*_1_′] for vehicle-, CGS21680-, and KW6002-treated rats.

Striatal BP_ND_ values obtained from SRTM revealed no significant differences between the CGS21680 or KW6002 pretreated rats and vehicle-treated rats (*F*(2,12) = 1.03, *p* = 0.38; Figure 6A). Additionally, there were no significant differences in *R*_1_ values between the three groups (*F*(2,12) = 0.07, *p* = 0.93; Figure 6B and Table S2).

### 3.3 Tracer Kinetics and Metabolism of [^11^C]raclopride in Whole Blood and Plasma

**Figure 7:**
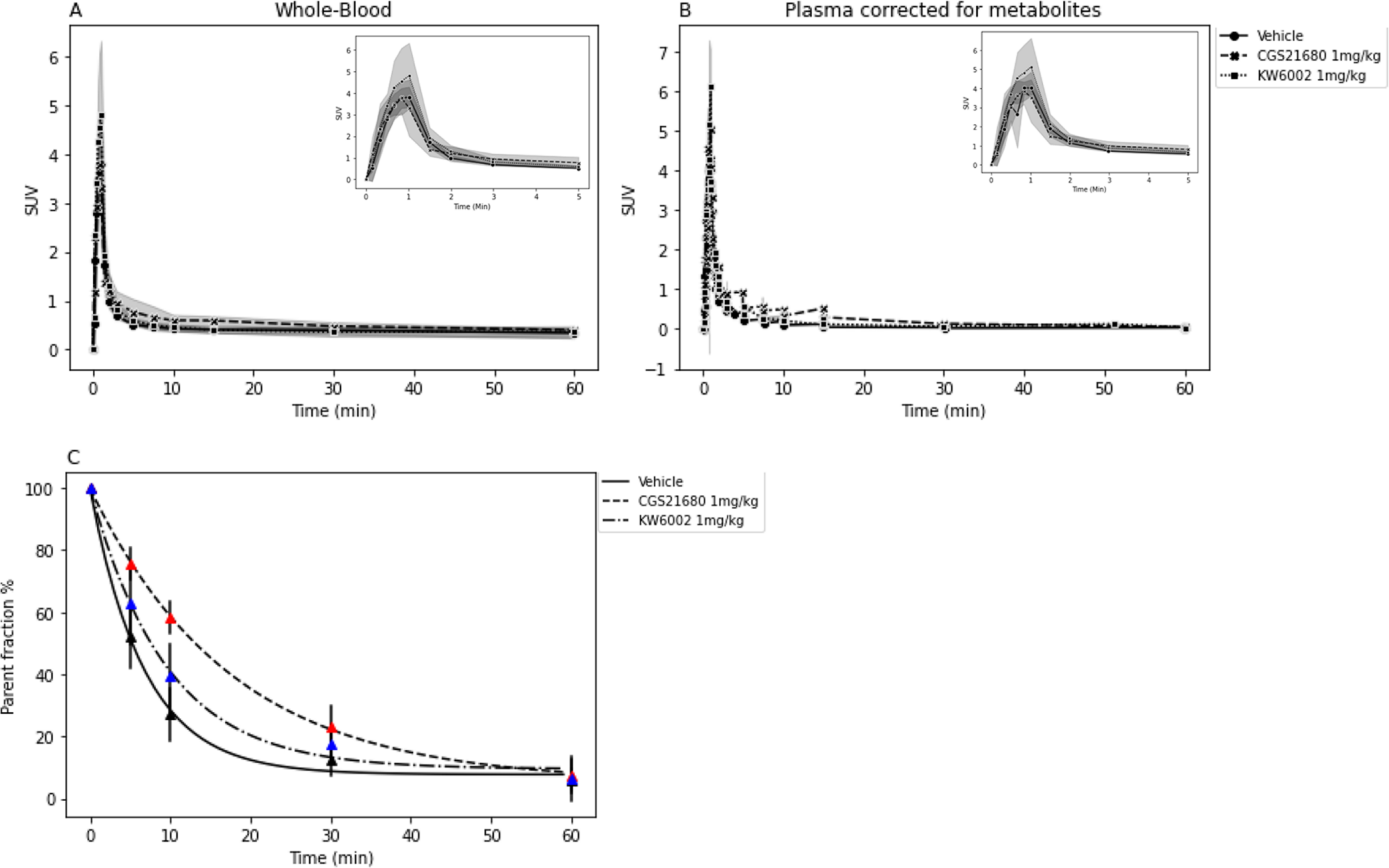
Time–activity curves of [^11^C]raclopride for scans with blood sampling. (A) Whole-blood and (B) metabolite-corrected plasma. (C) Exponential fit of the parent fraction of [^11^C]raclopride in plasma for rats pretreated with vehicle, CGS21680, or KW6002 (mean ± SD).

Figure 7A,B shows the plasma TACs corrected for metabolites and the whole-blood TACs during the 60 min dynamic scan. The AUC of whole-blood TACs was not significantly altered by pretreatment with CGS21680 (whole-blood Cohen’s *d* = 1.80) or with KW6002 (whole-blood Cohen’s *d* = 0.91) compared to vehicle-treated rats (whole blood, *F*(2,12) = 2.44, *p* = 0.129; Table 1). The AUC of the metabolite-corrected plasma TACs was significantly altered by pretreatment with CGS21680 (corrected plasma Cohen’s *d* = 2.3) and was not significantly altered by pretreatment with KW6002 (corrected plasma Cohen’s *d* = 1.3) compared to vehicle-treated rats (corrected plasma, *F*(2,12) = 5.3, *p* = 0.02; Table 1). The fraction of radioactivity in plasma consisting of intact [^11^C]raclopride was well described by an exponential function (Figure 7C). The parent fraction in plasma was significantly higher in the CGS21680-treated group (*F*(2,12) = 21.37, *p* = 0.0001) compared to vehicle, in particular at 10 and 30 min after tracer injection (*p* < 0.001; Table S3).

**Table 1:** AUC Values of Time–Activity Curves of Whole Blood- and Metabolite-Corrected Plasma^a^

group	whole blood AUC	Cohen’s *d*	*p*-value	metabolite-corrected plasma AUC	Cohen’s *d*	*p*-value
vehicle	25 ± 4.5			9.5 ± 2.0		
CGS21680 (1 mg/kg)	34.9 ± 6.3	1.8	0.06	17.3 ± 4.6*	2.2	0.64
KW6002 (1 mg/kg)	29.3 ± 4.9	0.9	0.69	13.2 ± 4.0	1.2	0.79

a Data are shown as mean ± SD. Statistically significant between-group differences compared to the vehicle group are indicated with asterisks; **p < 0.01. Cohen’s *d* is between the control and treatment groups.

### 3.4 Estimation of the Binding Potential Derived from 2TCM

Comparison of the BP_ND_ estimated from the *k*_3_/*k*_4_ ratio showed a significant difference between groups (*p* = 0.002). Post hoc analysis showed that the *k*_3_/*k*_4_ ratio in CGS21680-treated animals was significantly lower than that in vehicle-treated rats (*p* < 0.01, Cohen’s *d* = 3.66), whereas no effect of KW6002 was observed (*p* = 0.099; Cohen’s *d* = 1.04; Table 2).

**Table 2:** *k*_3_, *k*_4_, *k*_3_/*k*_4_, and *V*_T_ Values of Striatum, Caudate-Putamen, Globus Pallidus, Accumbens, and Cerebellum across Treatments, Determined with 2TCM^a^

treatment	kinetic parameters	striatum	caudate-putamen	globus pallidus	cerebellum	accumbens
vehicle	*k*_3_ (min^–1^)	0.138 ± 0.027	0.138 ± 0.026	0.158 ± 0.063	0.117 ± 0.032	0.055 ± 0.008
	*k*_4_ (min^–1^)	0.056 ± 0.009	0.056 ± 0.009	0.061 ± 0.013	0.055 ± 0.011	0.054 ± 0.013
	*k*_3_/*k*_4_	2.46 ± 0.33	2.47 ± 0.36	2.60 ± 0.80	2.11 ± 0.26	1.06 ± 0.23
	*V*_T_ (mL cm^–3^)	12.54 ± 0.99	12.74 ± 0.87	12.39 ± 2.18	10.50 ± 1.22	4.26 ± 0.55
CGS21680 1 mg/kg	*k*_3_ (min^–1^)	0.033 ± 0.013***	0.034 ± 0.014***	0.029 ± 0.017***	0.028 ± 0.012***	0.012 ± 0.006***
	*k*_4_ (min^–1^)	0.026 ± 0.009**	0.026 ± 0.009**	0.022 ± 0.021**	0.023 ± 0.010**	0.015 ± 0.008***
	*k*_3_/*k*_4_	1.27 ± 0.32**	1.28 ± 0.35**	1.63 ± 3.6*	1.19 ± 0.32**	0.87 ± 0.20
	*V*_T_ (mL cm^–3^)	17.67 ± 4.84	17.82 ± 4.71	19.77 ± 3.76	15.09 ± 4.66	7.08 ± 1.82**
KW6002 1 mg/kg	*k*_3_ (min^–1^)	0.085 ± 0.027**	0.086 ± 0.027**	0.087 ± 0.028*	0.072 ± 0.028	0.035 ± 0.015
	*k*_4_ (min^–1^)	0.043 ± 0.011	0.043 ± 0.012	0.048 ± 0.013	0.041 ± 0.011	0.039 ± 0.013
	*k*_3_/*k*_4_	2.00 ± 0.53	2.02 ± 0.52	1.86 ± 0.54	1.75 ± 0.52	0.91 ± 0.27
	*V*_T_ (mL cm^–3^)	13.90 ± 4.91	13.92 ± 4.67	15.89 ± 4.98	11.70 ± 5.22	4.67 ± 1.56

a Values are reported as mean ± SD. Statistically significant between-group differences compared to the vehicle group are indicated: ***p < 0.0001; **p < 0.01; and and *p < 0.05.

**Figure 8:**
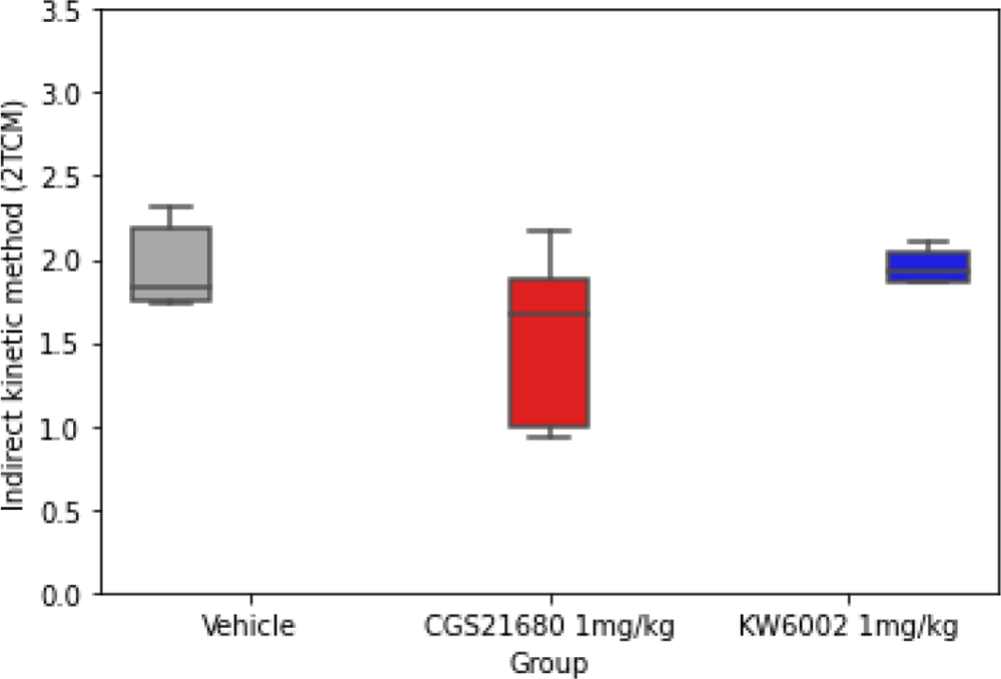
Indirect kinetic binding potential (BP_ND_) of [^11^C]raclopride in the striatum for the vehicle-, CGS21680-, and KW6002-treated rats determined with 2TCM.

The estimated striatal BP_ND_ obtained from DVR-1 was found to be the highest in the vehicle-treated rats (Figure 8; Table S2), with no significant differences between the treated groups (Table 3). Post hoc LSD (Fisher’s LSD) analysis of the outcome measures obtained for the striatum showed the highest value for the BP_ND_ (DVR-1) in vehicle-treated animals and the lowest value for the CGS21680-treated group. The differences between the vehicle-treated and CGS21680-treated and KW6002-treated groups were not statistically significant (*p* = 0.08, Cohen’s *d* = 0.99; *p* = 0.993, Cohen’s *d* = 0.01), although a large effect size was observed for the difference between the vehicle- and CGS21680-treated animals.

Simple correlation analysis (Table 3) showed good correlation between the BP_ND_ values estimated by the DVR-1 method and by SRTM for all groups (*r* = 0.996, *p* < 0.001; *r* = 0.934, *p* < 0.05; and *r* = 0.861, *p* = 0.061; Figure S4).

**Table 3:** BP_ND_ Values Estimated from 2TCM (DVR-1) and SRTM and Their Correlation

group	2TCM BP_ND_ (DVR-1)	SRTM BP_ND_	correlation
vehicle	1.963 ± 0.27	1.78 ± 0.23	0.99
CGS21680 1 mg/kg	1.53 ± 0.55	1.605 ± 0.29	0.93
KW6002 1 mg/kg	1.961 ± 0.106	1.74 ± 0.06	0.86

## 4 Discussion

Antagonistic interactions between adenosine A_2A_ and dopamine D_2_ receptors have been demonstrated in various in vitro systems. In membrane preparations from the rat striatum, the administration of the A_2A_R agonist CGS21680 decreases the affinity of dopamine D_2_R for the agonist L-(−)-[^3^H]NPA by 40%.^18^ Direct receptor–receptor interactions within neuronal membranes have been proposed as an explanation for such (and similar) findings.^18−20^

When human neuroblastoma (SH-SY5Y) cells transfected with human D_2_R are stimulated with CGS21680, the affinity of D_2_R in the cells to agonists is two- to threefold decreased.^21,22^ In Chinese hamster ovary (CHO) cells co-transfected with A_2A_R and D_2_R, stimulation of A_2A_R with CGS21680 results in a three- to fourfold decrease of the affinity of D_2_R for dopamine without any change of D_2_R numbers. A later study has shown that in CHO cells that are transiently transfected with A_2A_R and D_2_R, administration of either the A_2A_R agonist CGS21680 or the A_1_/A_2A_R antagonist caffeine reduces the affinity of D_2_R for radioligands, not only for the D_2_R agonist [^3^H]quinpirole but also for the D_2_R antagonist [^3^H]raclopride.^2^ In the striatal area of slices of rat and human brain, CGS21680 causes a significant increase of the IC_50_ values of competition between the D_2_R ligand [^125^I]iodosulpiride and dopamine.^6^

The primary objective of this study was to determine whether such A_2A_R–D_2_R interactions as have been reported in vitro can also be detected in vivo. Thus, we aimed to quantify D_2_ receptor availability in the striatum of healthy rats pretreated with A_2A_ ligands, using PET. As the cerebellum is a region with a negligible number of D_2_ receptors, it is often used as a reference region for kinetic modeling to estimate BP_ND_ values in [^11^C]raclopride PET studies.^15^ One such reference region method, SRTM, is a commonly used noninvasive analysis strategy for [^11^C]raclopride scans, as it does not require invasive and laborious arterial blood sampling. The BP_ND_ obtained via SRTM is generally known to provide similar sensitivity for detecting changes as the BP_ND_ estimated from DVR-1, using 2TCM with a metabolite-corrected arterial plasma input function. Thus, in our study, we initially applied this reference region method. Using SRTM, we could not detect any significant treatment-induced change in the striatal BP_ND_ of [^11^C]raclopride after the administration of CGS21680 (Figures 4 and 6).

Our drug treatments did not significantly affect the AUC of whole-blood TACs; however, the AUC of metabolite-corrected plasma TACs was significantly altered by pretreatment with CGS21680, indicating that CGS21680 may affect the tracer concentration in plasma. This proved indeed to be the case, as the fraction of plasma radioactivity representing metabolites was significantly affected by CGS21680 (Figure 7). The dose of CGS21680 that we applied in our study (1 mg/kg) also caused a strong reduction of heart rate, in some animals even down to 94 bpm, and the reduced heart rate persisted throughout the entire duration of the scan. Changes in tracer delivery in the CGS21680 group may thus also be related to changes in heart rate and blood pressure in the animals. Bolus administration of low doses of the adenosine receptor agonist NECA (5’-N-ethylcarboxamide adenosine has been shown to cause tachycardia, whereas a high dose of NECA (total dose of 3 mg/kg infused over 60 min) induces a rapid reduction in heart rate as an instant response.^23^ Previous studies have demonstrated the presence of A_2A_ receptors in porcine coronary arteries and rat thoracic aorta. These receptors, when stimulated by adenosine analogues, produce relaxation of vascular smooth muscles which can result in a drop of blood pressure.^24^ A high adenosine agonist and antagonist dose in combination with [^11^C]raclopride imaging has not been administered in the past. In the current study, we observe dramatic changes in the physiology of the CGS21680-treated animals. For this reason, a second group of animals was scanned with arterial blood sampling, and the PET data of this group were analyzed using the gold standard of compartmental modeling with the metabolite-corrected input function to quantify the binding of the radioligand to its target.

When the BP_ND_ was estimated from the *k*_3_/*k*_4_ ratio, a significant reduction in striatal tracer binding was observed in the CGS2160-treated group but not in the KW6002-treated group. However, the *k*_3_/*k*_4_ ratio tends to show a higher inaccuracy presumably due to the noise in the data; therefore, we considered these BP_ND_ estimates less reliable. When BP_ND_ was estimated from DVR-1, a bias of 10% was observed between SRTM and 2TCM values in the striatum, which is the highest binding region for [^11^C]raclopride. In basal ganglia, BP_ND_ values estimated with SRTM were well correlated, although not the same values, as those estimated by the indirect kinetic method with 2TCM. The estimates of BP_ND_ were slightly lower for the SRTM approach compared to the DVR-1 method using arterial sampling. Other investigators have also observed that the estimates of BP_ND_ (or DVR-1) were higher for the compartment model approach than for graphical methods using a reference tissue.^25^ Administration of a specific A_2A_ agonist (CGS21680) and antagonist (istradefylline, KW6002) did not cause a significant reduction in DVR-1, although the effect size was high between the vehicle- and CGS21680-treated animals (Table S2). Although a lower estimate of indirect estimate of binding potential was observed with the CGS21680-treated group compared to the vehicle, the differences were not significant due to interindividual variability. A power analysis calculation with DVR-1 values from the vehicle- and CGS21680-treated groups revealed that a group size of 17 animals was required to obtain a significant difference (Table S2). Additionally, we did not observe any significant differences in *V*_T_ measured from 2TCM in the striatum.

The trend toward a decrease in D_2_R availability induced by CGS21680 is in line with the previous in vitro studies that were discussed above^18,21−26^ and supports the hypothesis of direct A_2A_R–D_2_R interactions in the mammalian striatum.^19,20^ Experiments using electrically stimulated brain slices and microdialysis in intact rats have demonstrated that A_2A_R stimulation affects the levels of extracellular dopamine in the rodent striatum. Low doses of CGS21680 (0.01–1 μM) inhibit electrically evoked dopamine release,^25^ but locally administered higher doses (3, 10, 50, and 100 μM) of CGS21680 increase dopamine release in the entire striatum and the shell of the nucleus accumbens.^27^ Consequently, reduced [^11^C]raclopride binding may thus be due to the reduced affinity of the D_2_R-binding site in A_2A_R–D_2_R heteromeric complexes for the tracer or increased competition by endogenous dopamine for tracer binding, due to increased dopamine release.

After the administration of the specific A_2A_R antagonist istradefylline (KW6002), the binding potential of [^11^C]raclopride in the striatum was not significantly affected. A previous PET study has applied a similar paradigm in humans. In that study, oral caffeine (300 mg) was administered to healthy subjects with low levels of daily caffeine intake, and changes of the binding of [^11^C]raclopride to D_2_R were assessed. Caffeine was found to cause a slight (5–6%) increase of D_2_R/D_3_R availability in the putamen and ventral striatum but not the caudate nucleus of the human brain.^28^ The increase of D_2_R availability in the ventral striatum was related to the increase of alertness that was induced by caffeine. The findings of this human study seem to contradict our results on rodents, but for several reasons, the results of the two studies cannot be directly compared. Caffeine is an antagonist at all four subtypes of adenosine receptors (A_1_, A_2A_, A_2B_, and A_3_), whereas KW6002 is considered as an A_2A_R-selective antagonist with much higher affinity for A_2A_R than caffeine. Thus, caffeine may have a more complex mechanism of action and different effects than KW6002. The human PET study used a ratio method for data analysis in which cerebellum was used as a reference tissue for the estimation of the tracer binding potential in the target areas of the brain, whereas our study used a two-tissue compartment model fit with the metabolite-corrected plasma data as the input function. Thus, different methods for data analysis were applied in both studies. Finally, species differences between humans and rodents may have resulted in a different outcome.

## 5 Conclusions

The present study suggests that the administration of an adenosine A_2A_ agonist may cause a slight reduction of the binding potential of a dopamine D_2_ ligand in the striatum of living rats. However, due to considerable interindividual variability, larger groups of animals would be required for this effect to reach statistical significance (much larger than the group size of 5 that was used here). The binding potentials from SRTM and the DVR-1 method were well correlated, although the use of SRTM provided bias with lower values in high-binding regions. BP_ND_ values derived from the *k*_3_/*k*_4_ ratio did show a significant reduction in tracer binding after CGFS21680 administration, but these results were considered less reliable due to the sensitivity to noise.

## Supporting Information Available

AUC of time–activity curves of striatum and cerebellum with and without arterial blood sampling; BP_ND_, indirect BP_ND_, and *R*_1_ values derived from the baseline and follow-up scans of animals pretreated with vehicle, CGS21680, and KW6002; percentage parent tracer in plasma; correlation of BP_ND_ values estimated by indirect kinetic method using parent as an input function and by the SRTM method (PDF)
