# Supplementary material for: Impact of an Adenosine A2A Receptor Agonist and Antagonist on Binding of the Dopamine D2 Receptor Ligand [11C]raclopride in the Rodent Striatum
Source: Mol Pharm. 2022 Jul 18;19(8):2992–3001. doi: 10.1021/acs.molpharmaceut.2c00450 (PMC9346611; doi:10.1021/acs.molpharmaceut.2c00450)
Supplement: Supplementary file 1 — mp2c00450_si_001.pdf [file mp2c00450_si_001.pdf]

Supporting Information

S.1 Supplemental table 1: AUC of time-activity-curves of striatum and cerebellum from scans with and without arterial blood sampling. Data is shown as mean±SD

**Scan (Without sampling)**	**Striatum**	**Cohen's *d***	**Cerebellum**	**Cohen's *d***
**Baseline**	84±13		36±5	
**Follow-up (CGS21680 1mg/kg)**	138±17	3.81	58±9	3.02
**Scan (With sampling)**				
**Vehicle**	89±15		33±5	
**CGS21680 (1mg/kg)**	135±55	1.15	77±18	3.41
**KW6002 (1mg/kg)**	129±39	1.74	50±16	0.10

S.2 Supplemental table 2: BP_{ND} , indirect BP_{ND} and R_1 values derived from baseline and follow-up scans of animals pre-treated with vehicle, CGS21680 and KW6002. Data is shown as mean±SD.

**Scan (Without sampling)**	***BP_{ND}* (SRTM)**	**Cohen's *d***		**R_1**	**Cohen's *d***			
**Baseline**	1.47±0.24			0.99±0.07				
**Follow-up (CGS21680 1mg/kg)**	1.67±0.11	0.63		1.10±0.10	0.82			
**Scan (With sampling)**	***BP_{ND}* (SRTM)**	**Cohen's *d***	**Power calculation**	**R_1**	**Cohen's *d***	**2TCM BP_{ND} (DVR-1)**	**Cohen's *d***	**Power calculation**
**Vehicle**	1.78±0.10			1.13±0.11		1.963±0.27		
**CGS21680 (1mg/kg)**	1.60±0.29	0.13	24	1.12±0.10	0.08	1.530±0.55	0.99	17
**KW6002 (1mg/kg)**	1.77±0.06	0.028	1069	1.14±0.03	0.15	1.961±0.11	0.01	166791

S.3 Supplemental table 3. Percentage parent tracer in plasma. Data is shown as mean±SD. Statistically significant between-group differences compared to the vehicle group as indicated **p<0.01 and *p<0.05.

**Time [Min]**	**Vehicle**	**CGS21680 (1mg/kg)**	**Cohen's *d***	**KW6002 (1mg/kg)**	**Cohen's *d***
**0**	100±0	100±0		100±0	
**5**	59.3±2.1	74.3±6.4	3.2	62.7±13.1	0.4
**10**	27.2±8.5	**58.3±5.1****	4.4	39.5±10.1	1.1
**30**	12.3±5.0	**23.4±6.3***	1.9	17.4±6.5	0.9
**60**	5.8±3.8	7.6±4.7	0.4	6.5±7.1	0.1

S.4.

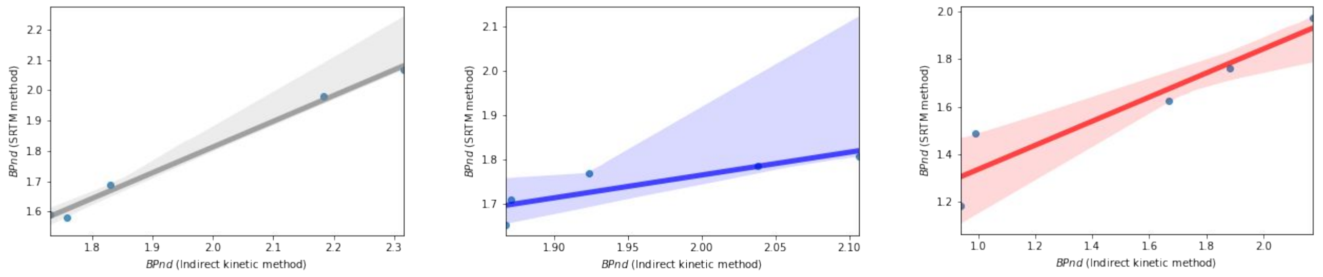

Supplemental figure 1. Correlation of BP_{ND} values estimated by indirect kinetic method using parent as input function and by SRTM method. BP_{ND} values showed significant correlation between two methods for Vehicle and CGS21680 treated.
